# Is fatigue associated with adolescents’ mental health? A pre- vs. post-COVID-19 cross-sectional survey comparison

**DOI:** 10.3389/frcha.2026.1745757

**Published:** 2026-07-22

**Authors:** Anne Abio, Andre Sourander, Omid Dadras, Susanna Hinkka-Yli-Salomäki, Sonja Gilbert, Olli Ruuskanen

**Affiliations:** 1 Department of Child Psychiatry, Research Centre for Child Psychiatry, University of Turku, Turku, Finland; 2 INVEST Research Flagship Center, University of Turku, Turku, Finland; 3 Department of Child Psychiatry, Turku University Hospital, Turku, Finland; 4 Department of Paediatrics and Adolescent Medicine, Turku University Hospital, University of Turku, Turku, Finland

**Keywords:** adolescents, Finland, mental health, psychopathology, time-trend study

## Abstract

**Aim:**

Fatigue is a common complaint reported by adolescents and adults; and one of the persistent symptoms of long COVID-19. The aim of this study was to assess the prevalence of self-reported fatigue and associated factors among adolescents in Finland at two distinct time points, pre- (2018) and post-COVID (2023).

**Methods:**

A cross-sectional survey was conducted among adolescents in two cities, Rovaniemi and Salo, Finland, in 2018 and 2023. All schools with students in the 7–9th grades in Rovaniemi (10) and Salo (4) cities were invited to participate in the survey, except special needs schools and classes. The Checklist Individual Strength (CIS) questionnaire was used to determine the severity of fatigue among adolescents. A total of 5,583 adolescents aged 13–17 years (*n* = 2,774, in 2018) and (*n* = 2,809, in 2023) were included in the analyses. Linear regression analysis was used to determine the association between fatigue, survey year and explanatory variables.

**Results:**

The prevalence of fatigue increased among females from 16.2% in 2018 to 24.3% in 2023 (*p* < 0.001), but not among males at 6.7% vs. 5.6% (*p* = 0.239) respectively. The mean fatigue score change among females was 29.1 vs. 32.2 (*p* < 0.001), and males 24.6 vs. 25.2 (*p* = 0.081) in 2018 vs. 2023 respectively. The fatigue score was higher among females by 0.7 points in 2023 (*β* = 0.68; 95% CI: 0.07, 1.28), but not among males (*β* = 0.03; 95% CI: −0.56, 0.62). Internalizing problems were associated with fatigue among females (*β* = 5.00; 95% CI: 4.68, 5.32); and males (*β* = 3.74; 95% CI: 3.37, 4.11). Externalizing problems were associated with fatigue (*β* = 2.87; 95% CI: 2.53, 3.20) among females and (*β* = 2.19; 95% CI: 1.85, 2.53) males. Other factors associated with fatigue included living with one biological parent and being overweight among across both sexes.

**Conclusion:**

The notable increase in fatigue, among girls, post-COVID highlights the need for sustained attention to adolescent mental health and physical health, especially in relation to internalizing and externalizing problems.

## Summary

COVID-19 caused significant changes globally, including lifestyle among adolescents. This study found self-reported fatigue increased among female adolescents in Finland utilizing cross-sectional surveys conducted before (2018) and after the COVID-19 pandemic (2023). Higher levels of internalizing and externalizing problems were associated with higher levels fatigue across both sexes.

## Introduction

Fatigue is a common complaint among both adolescents and adults (1–4), characterized by tiredness, lack of energy, weakness and exhaustion (1, 3, 5, 6). While fatigue can be transient, it is also a prominent feature of several chronic illnesses including sleep disorders, cardiovascular and pulmonary conditions, neurological diseases, depression, cancer, autoimmune diseases, and sequalae from trauma like head injuries, among others (1, 5, 6). In addition, fatigue may result from viral infections, such as COVID-19, where it has emerged as a hallmark of long COVID (7, 8). Chronic Fatigue Syndrome/myalgic encephalitis (CFS/ME) includes the debilitating chronic and unexplained fatigue experienced by an individual over a prolonged period of time that overlaps with other symptoms like sleep problems, musculoskeletal pain, and concentration or short-term memory problems (9).

Fatigue in adolescents is of particular concern and rarely occurs in isolation; instead, it may affect education outcomes but could be interwoven with broader emotional and behavioural difficulties. For example, it can be attributed to factors such as, psychological stress, lifestyle changes, hormonal fluctuation, social demands, educational pressures, school attendance and performance contributing to absenteeism (4). A systematic review focusing on long COVID-19 symptoms reported pre-COVID (1989–2019) found a pooled prevalence of 20.5% of fatigue from 4 studies among adolescents aged 10–19 years (10). A third of adolescents aged 11–17 years reported morning fatigue occurring at least four times in a week in Europe in 1997/1998 (11). Fatigue among adolescents is an important symptom, given its potential to impair both physical and mental well-being. Prevalence rates among adolescents in the general population vary widely, ranging from 7% to 38% in studies in the Netherlands, United Kingdom, United States, and Japan with higher rates reported among girls (2, 4, 12, 13). Furthermore, a prevalence ranging from 7% to 45% has been reported in the adult population (1, 9, 14, 15). Community studies show that youth who report higher fatigue levels also exhibit elevated internalizing symptoms, such as anxiety, depression, and somatic complaints, as well as externalizing behaviours including conduct problems and hyperactivity (16, 17). Clinical samples further demonstrate that sleep-related fatigue predicts both mood disturbance and behavioural dysregulation in adolescents seeking mental health services (17). Moreover, emerging research on long COVID indicates that persistent fatigue among adolescents often co-exists with anxiety, low mood, and irritability, suggesting that post-infection sequelae may exacerbate underlying psychological vulnerabilities (18, 19).

Fatigue can exist along a continuum, ranging from mild, intermittent tiredness to chronic, debilitating exhaustion and occur seasonally (1). This variability in the presentation of fatigue underscores the challenge of measuring and defining it consistently. Although several validated self-report scales exist, there is no universal consensus on a singular definition of fatigue (1, 5). Most studies focus on patient populations with chronic illnesses, with relatively few examining fatigue symptoms within the general population, more so among adolescents (20–23). Even fewer studies have examined how fatigue levels change over time, particularly in relation to major societal disruptions such as the COVID-19 pandemic. The COVID-19 pandemic introduced unprecedented changes in adolescent lifestyles, including prolonged social isolation, disruption of routines, and heightened psychological stress; which may have contributed to increased fatigue (24, 25). Despite these concerns, no study to date has directly assessed adolescent fatigue levels both before and after the pandemic. Given the emerging recognition of long COVID symptoms and growing concern for adolescent mental health, understanding how fatigue has evolved in this age group is both timely and essential. Repeated cross-sectional surveys conducted using the same methodological design at different time points allow for comparisons across time and are suitable for estimating temporal changes in wellbeing within the general population.

This study is part of the Finnish time trend studies utilizing cross sectional surveys conducted in two cities in 1998, 2008, 2014, 2018 and 2023 (26–32). Rovaniemi is located in the Artic Circle in the northern region, while Salo is located in the southern part of Finland. Both cities were selected owing to their geographic and demographic representativeness that reflect national averages in Finland (33). The surveys use a similar methodological design and focus on adolescent health and well-being. Fatigue affects health and well-being, and was added as theme for investigation from 2018. This study seeks to fill this gap by estimating and comparing the severity of fatigue among Finnish adolescents at two time points: pre-pandemic (2018) and post-pandemic (2023).

## Methods

### Subjects and procedure

The study used cross-sectional surveys conducted in 2018 and 2023 in two cities in Finland, Rovaniemi and Salo. Due similar methodological designs of the surveys, comparison of changes in fatigue symptoms across time was possible. The target population were all students in the 7–9th grades enrolled in Salo and Rovaniemi school districts. All adolescents in grades 7, 8, and 9 (typically aged 13–17 years) from all secondary schools in both cities were invited to participate in the study, except schools and classes for children with special needs. All four and ten schools in Salo and Rovaniemi were invited to participate in both years respectively. All the schools in Salo agreed to participate at both times. In Rovaniemi, one school declined to participate in 2018, while two schools declined in 2023 due to other ongoing priorities at the time. Data collection was conducted from April to May in 2018 and March to April in 2023.

The sex distribution, ethnic backgrounds and family compositions in both cities are comparable to the national demographics for the study age group according to Finnish Statistics (33). Rovaniemi and Salo were chosen as study locations due to their representativeness of both the northern (Rovaniemi) and southern (Salo) regions of Finland. Rovaniemi and Salo have a population of approximately 64,000 and 50,000 inhabitants respectively, providing a balanced sample from both geographical and demographic perspectives. This regional diversity adds a broader scope to the findings and ensures that the results reflect the varying living conditions of adolescents in Finland.

**Figure 1:**
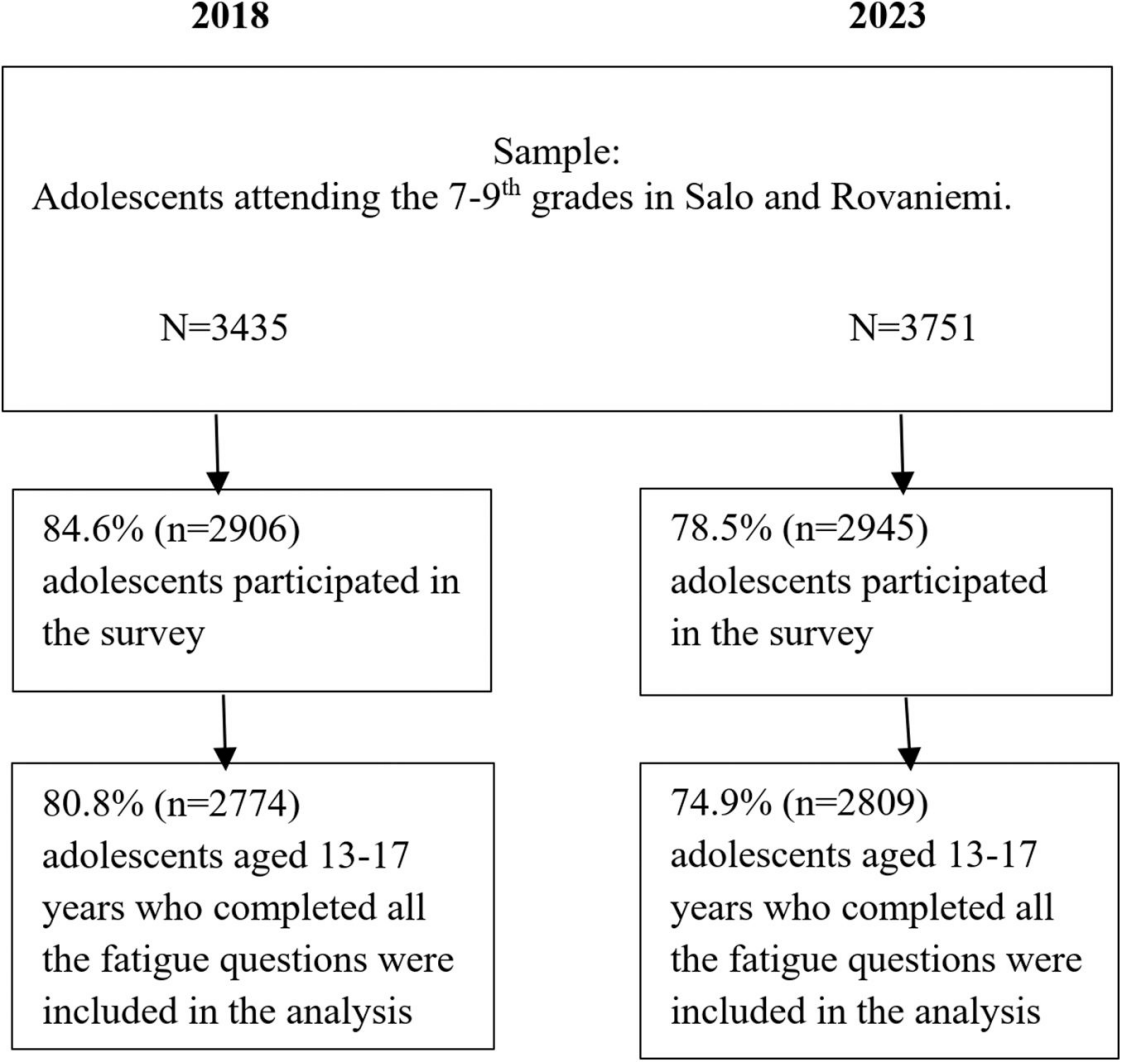
Participation rates among adolescents aged 13–17 years.

The surveys were administered during regular classroom sessions, and participation was voluntary. Adolescents present in school on the day of the survey were invited to participate voluntarily in the study with informed consent. The questionnaire was distributed to adolescents during a normal class lesson. The questionnaire was filled out anonymously and returned to the teacher in a sealed envelope. The sealed envelopes were then placed by the teacher into a bigger envelope and sealed in the presence of the students and returned to the researchers. Students who were absent on the day of the survey also had the opportunity to fill in the questionnaire later. A total of 3,435 in 2018 and 3,751 students in 2023 were invited to participate in the survey. Students who declined to participate were 529 in 2018 and 806 in 2023. Among the respondents, 13 and 42 questionnaires in 2018 and 2023 respectively were excluded due to incomplete or inappropriate answers (e.g., if a questionnaire consisted only or mostly of swear words or inappropriate drawings). A total of 2,774 (80.0%) in 2018 and 2,809 (74.9%) in 2023 study participants with complete data on fatigue symptoms were included in the analyses. A flow chart illustrating the final sample size and participant inclusion process for each year is provided in Figure 1. Ethical approval for both surveys was obtained from the Ethical Committee of the University of Turku in 2018 (IRB No 64/2017) and 2023 (IRB No 10/2023), and permission was sought from the respective school authorities. The confidentiality and anonymity of the adolescents was ensured during the entire research study. As per the Finnish regulations, parental consent was not required; however, parents were informed about the research study and could request their children not to participate (34).

### Measures and variables

#### Demographic information

Demographic information collected included age, sex, place of residence, family structure and family background. Age was used as a categorical variable (13 vs. 14 vs. 15 vs. 16–17 years). City of residence was categorized as Salo vs. Rovaniemi. The place of birth was categorized as born in Finland vs. abroad. Body Mass Index was categorized as normal vs. underweight vs. overweight and based on the standard criteria for Finnish children (35). The family structure was categorized into three groups: living with both biological parents, vs. one biological parent vs. others for example, foster parents, adoptive parents, relatives. Foster parents were adults who act as caregivers or caretakers of children from the child protection services office. These factors were considered important covariates due to previous research. Sociodemographic factors such as being overweight may be linked to fatigue and mental health (36). A Finnish study found seasonal mood and behavioral variation in the north vs. south which highlights the need to explore fatigue symptoms by region (31).

### Fatigue

Fatigue was self-reported and measured using the Checklist Individual Strength (CIS) questionnaire, which has been widely validated for assessing fatigue in clinical and general populations, including adolescents in Netherlands (5, 23). The CIS was selected for this study due to its robust psychometric properties, including high reliability and validity, and its ability to capture multiple dimensions of fatigue, making it ideal for use in both clinical and general populations (5, 23). The questionnaire consists of 4 subscales: severity of fatigue, concentration, motivation and physical activity. For this study, we focused on the severity of fatigue subscale which comprised of 8 statements. The items included: 1) I feel tired, 2) I feel physically tired, 3) I feel fit, 4) I feel powerless, 5) I feel well rested, 6) I feel out of shape, 7) I get tired easily, and 8) I feel very fit. These items were scored on a 7-point Likert scale (“1 = yes, that is true”, to “7 = no, that is not true”). Adolescents were requested to describe how they felt during the past 2 weeks in response to the statements in the questionnaire.

The total fatigue score for each participant was calculated by summing the responses, with the positive statements scored in reverse. The total scores range from 8 to 56, with higher scores indicating greater fatigue severity. This total fatigue score served as the primary outcome variable in the analyses. To determine the change in prevalence of fatigue symptoms, we used a cut-off based on the 90th percentile, with 2018 as the base year.

### Mental health

Internalizing and externalizing problems were assessed using the Strengths and Difficulties Questionnaire (SDQ). The questionnaire includes 25 items with three response choices “not true”, “somewhat true” and “certainly true” and scores ranging from 0 to 2. The SDQ tool is available from YouthinMind (37) and has been validated in an adolescent sample in Finland (27). The items are divided into 5 subscales: emotional symptoms, conduct problems, hyperactivity, peer problems, and prosocial behavior. The positively worded statements were scored in reverse. The total subscale scores range from 0 to 10, with higher scores indicating more problems. Internalizing problems was derived from summing emotional and peer problems (range 0–20), while conduct problems and hyperactivity were summed up for externalizing problems (range 0–20) (38). Similar to the subscales, higher scores mean more problems. Internalizing and externalizing symptoms were included as covariates to control for the effect of mental health, due to co-occurrence with fatigue (16, 17).

### Statistical analysis

Categorical variables are presented using counts and percentages with differences between groups assessed using Pearson's chi-square tests. Continuous variables were presented with the means, standard deviation, while differences were tested using the Student's *t*-test because the same individuals did not participate in the survey in 2018 and 2023. The association between fatigue and covariates was examined using linear regression analysis.

The analysis was stratified by sex. Significant interactions were found between year and sex (*F* = 23.4, *p* < 0.001) highlighting the need for sex-specific analyses. For the adjusted models, age, city of residence, family structure, birthplace, and psychopathology in the form of externalizing and internalizing problems were used as covariates. Internalizing and externalizing problems variables were standardized with a standard deviation of 1 by rescaling each variable to have a mean of zero and a standard deviation of one for each sex separately. Three models were used in the analyses: Model 1 was adjusted for the survey year only; Model 2 included the survey year and adjusted for sociodemographic variables (age, city, family structure, birthplace) to control for their effect on fatigue. Model 3 included the survey year, and adjusted for sociodemographic and mental health variables (age, city, family structure, birthplace, internalizing problems and externalizing problems). Mental health symptoms were adjusted for Model 3 to account for their effect based on co-occurrence with fatigue. We used robust standard errors to control for heteroskedasticity in all models. The fitness of the models were tested and found to have a good fit. Collinearity was tested using the Variance Inflation Factor (VIF) and found to be minimal with values ranging from 1.0 to 1.8. Statistical significance was set at *p* < 0.05 with a corresponding 95% confidence interval. Data analyses were conducted using Stata 18 (StataCorp, TX, USA).

## Results

The background characteristics of the study participants in 2018 and 2023 are presented in Table 1. Overall, the characteristics remained consistent across both years. Among females, the distribution remained stable, with 32% aged 14 years (*p* = 0.74), family composition with 72% vs. 75% living with two biological parents (*p* = 0.18), 96% were born in Finland (*p* = 0.74) and normal weight BMI was reported by 82% (0.39). Mental health symptom scores (internalizing and externalizing problems) increased in 2023 (*p* < 0.001). Among boys, the proportion aged 16–17 years reduced from 15% to 11% in 2023 (*p* = 0.003), while the proportion residing in Rovaniemi increased from 47% to 51% (*p* = 0.03). Family structure composition (*p* = 0.41), place of birth (*p* = 0.79) and BMI (*p* = 0.54) remained consistent. For mental health, only externalizing symptom scores increased in 2023 (*p* < 0.001).

**Table 1:** Background characteristics and SDQ scores of study participants.

Variable	Females (*N* = 2,689)			Males (*N* = 2,751)		
	2018	2023	Chi/*t*-test *p*-value	2018	2023	Chi/*t*-test *p*-value
Age *n* (%)			0.74			0.003
13	294 (21.4)	295 (22.8)		259 (18.9)	296 (21.8)	
14	443 (32.3)	417 (32.3)		440 (32.1)	470 (34.7)	
15	462 (33.7)	431 (33.3)		462 (33.7)	442 (32.6)	
16–17	174 (12.7)	150 (11.6)		209 (15.3)	148 (10.9)	
City *n* (%)			0.08			0.03
Salo	727 (52.8)	647 (49.4)		728 (52.9)	669 (48.7)	
Rovaniemi	651 (47.2)	664 (50.7)		649 (47.1)	705 (51.3)	
Family structure *n* (%)			0.18			0.41
Two biological parents	981 (72.0)	976 (74.7)		1,041 (76.2)	1,047 (76.5)	
Remarried/one biological parents/same sex	352 (25.8)	310 (23.4)		291 (21.3)	297 (21.7)	
Foster/adoptive/other parents	30 (2.2)	20 (1.5)		34 (2.5)	24 (1.8)	
Birth place *n* (%)			0.74			0.79
Born in Finland	1,318 (95.9)	1,260 (96.1)		1,312 (96.1)	1,315 (95.9)	
Born abroad	57 (4.2)	51 (3.9)		54 (4.0)	57 (4.2)	
BMI* *n* (%)			0.39			0.54
Normal	1,036 (81.6)	923 (82.3)		913 (71.1)	861 (70.8)	
Underweight	53 (4.2)	35 (3.1)		39 (3.0)	29 (2.4)	
Overweight	181 (14.3)	164 (14.6)		332 (25.9)	327 (26.9)	
Psychopathology						
Mean (SD)						
Internalizing	6.6 (3.5)	7.4 (3.5)	<0.001	4.2 (3.2)	4.4 (3.2)	0.22
Range	0–18	0–18		0–20	0–18	
Externalizing	5.6 (3.3)	7.1 (3.9)	<0.001	5.5 (3.1)	6.1 (3.4)	<0.001
Range	0–17	0–19		0–17	0–20	

* BMI categorization: Girls (Underweight <16.1, Normal 16.1–24.0, Overweight >24.0); Boys (Underweight <15.9, Normal 15.9–22.4, Overweight >22.4).

### Females

**Figure 2:**
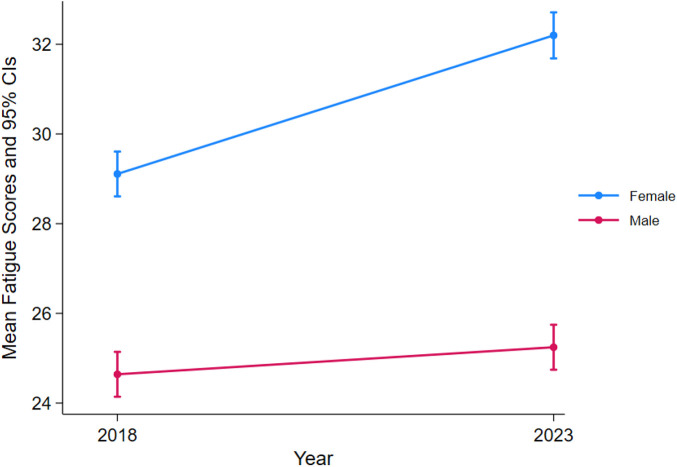
Mean fatigue scores in 2018 and 2023 among adolescents aged 13–17 years: by sex.

The mean fatigue score change pre- and post-COVID was 29.1 and 32.2 (*p* < 0.001) as shown in Figure 2. Approximately 16.2% (*n* = 223) and 24.3% (*n* = 319) (*p* < 0.001) of females were in the 90th percentile of fatigue in 2018 and 2023 respectively. This represents a rise in fatigue symptoms among adolescent females post-COVID.

In model 1, fatigue was higher by 3.1 points in 2023 (*β* = 3.09; 95% CI: 2.34, 3.84) as shown in Table 2. After adjusting for year, girls aged 16–17 years had fatigue scores higher by 3 points compared to 13-year-olds (*β* = 3.15; 95% CI: 1.88, 4.41). Girls living with foster or adoptive parents reported 5.6 higher fatigue scores compared to those living with two biological parents (*β* = 5.63; 95% CI: 3.06, 8.20). Compared to normal BMI, underweight girls had lower fatigue scores (*β* = −3.98; 95% CI: −6.17, −1.79), while overweight girls had higher fatigue scores (*β* = 3.60; 95% CI: 2.50, 4.70). Internalizing (*β* = 6.03; 95% CI: 5.73, 6.32) and externalizing problems (*β* = 4.59; 95% CI: 4.25, 4.94) were associated with higher fatigue scores.

**Table 2:** Association of fatigue symptoms and explanatory variables among females.

*N* = 2,689	Mean distribution	Model 1			Model 2			Model 3		
Variable	Mean fatigue score	*β* coef	95% CI	*p*-value	*β* coef	95% CI	*p*-value	*β* coef	95% CI	*p*-value
Year										
2018	29.1	Ref			Ref			Ref		
2023	32.2	3.09	2.34, 3.84	<0.001	3.14	2.37, 3.92	<0.001	0.68	0.07, 1.28	0.03
Age										
13	28.9	Ref			Ref			Ref		
14	30.5	1.61	0.57, 2.66	0.003	1.52	0.41, 2.64	0.01	0.91	0.10, 1.73	0.03
15	31.5	2.64	1.62, 3.65	<0.001	1.95	0.87, 3.03	<0.001	1.65	0.83, 2.47	<0.001
16–17	31.9	3.15	1.88, 4.41	<0.001	2.35	0.98, 3.72	0.001	1.50	0.48, 2.53	0.004
City										
Salo	30.8	Ref			Ref			Ref		
Rovaniemi	30.4	−0.55	−1.30, 0.20	0.15	−0.58	−1.36, 0.20	0.144	−0.79	−1.38, −0.20	0.01
Family structure										
Two biological parents	29.7	Ref			Ref			Ref		
One biological/remarried parent/same sex	33.0	3.36	2.49, 4.22	<0.001	3.32	2.41, 4.24	<0.001	0.84	0.13, 1.55	0.02
Foster/adoptive/other parents	35.0	5.63	3.06, 8.20	<0.001	5.81	3.17, 8.45	<0.001	0.73	−1.38, 2.84	0.50
Birth place										
Born in Finland	30.6	Ref			Ref			Ref		
Born abroad	30.0	−0.55	−2.32, 1.21	0.54	−1.93	−3.72, −0.13	0.04	−1.06	−2.48, 0.35	0.14
BMI^†^										
Normal	30.0	Ref			Ref			Ref		
Underweight	25.8	−3.98	−6.17, −1.79	<0.001	−3.39	−5.54, −1.24	0.002	−2.10	−3.67, −0.54	0.01
Overweight	33.6	3.60	2.50, 4.70	<0.001	3.21	2.11, 4.31	<0.001	1.17	0.30, 2.03	0.01
Psychopathology^‡^										
Internalizing problems		6.03	5.73, 6.32	<0.001	—	—	—	5.00	4.68, 5.32	<0.001
Externalizing problems		4.59	4.25, 4.94	<0.001	—	—	—	2.87	2.53, 3.20	<0.001

† Body Mass Index (BMI) categorization: Girls (Underweight <16.1, Normal 16.1–24.0, Overweight >24.0).

‡ Psychopathology: internalizing and externalizing standardized with a standard deviation of 1.

Model 1: Adjusted for year.

Model 2: Adjusted for year, age, city, family structure, birthplace, BMI.

Model 3: Adjusted for year, age, city, family structure, birthplace, BMI, internalizing problems, externalizing problems.

Missing numbers by variable: fatigue 0; year 0; age 23; city 0; BMI 297; family structure 20; birth place 3; internalizing 5; externalizing 4.

After adjusting for sociodemographic variables (Model 2), fatigue scores remained higher by 3.1 points in 2023 (*β* = 3.14; 95% CI: 2.37, 3.92). Girls aged 16–17 years had the highest fatigue scores in comparison to 13-year-olds (*β* = 2.35; 95% CI: 0.98, 3.72). Girls living with foster/adoptive parents had higher fatigue scores (*β* = 5.81; 95% CI: 3.17, 8.45), while those born abroad had lower scores (*β* = −1.93; 95% CI: −3.72, −0.13). Congruently, being underweight was associated with lower fatigue scores (*β* = −3.39; 95% CI: −5.54, −1.24), and overweight with higher scores (*β* = 3.21; 95% CI: 2.11, 4.31).

In Model 3, after adjusting for sociodemographic and the mental health variables, the fatigue score was higher by 0.7 points in 2023 (*β* = 0.68; 95% CI: 0.07, 1.28). Fifteen-year-old girls had higher fatigue scores (*β* = 1.65; 95% CI: 0.83, 2.47). Girls living with one biological parent or remarried biological parent had 0.8 higher fatigue scores compared to those living with two biological parents (*β* = 0.84; 95% CI: 0.13, 1.55). A one-unit increase in the standardized internalizing problem score was associated with a 5-point higher fatigue score (*β* = 5.00; 95% CI: 4.68, 5.32); while externalizing problems was (*β* = 2.87; 95% CI: 2.53, 3.20).

### Males

The mean fatigue score pre and post-COVID was 24.6 and 25.2 (*p* = 0.081). Among males, 6.7% (*n* = 92) were in the 90th percentile of fatigue in 2018, and 5.6% (*n* = 77) in 2023 (*p* = 0.239); which represented a stable pattern of fatigue symptoms pre and post-COVID-19.

In Model 1, there was no significant increase in fatigue scores in 2023 (*β* = 0.60; 95% CI: −0.07, 1.28). Adjusting for year, boys aged 16–17 years had higher fatigue compared to 13-year-olds (*β* = 1.34; 95% CI: 0.20, 2.48). Living in Rovaniemi was associated with lower fatigue (*β* = −1.53; 95% CI: −2.20, −0.85). Boys living with foster or adoptive parents had higher fatigue compared to those living with two parents (*β* = 3.21; 95% CI: 0.83, 5.59). Overweight boys had 3.5 higher fatigue scores compared normal weight boys (*β* = 3.53; 95% CI: 2.73, 4.33). Internalizing and externalizing problems were associated with higher fatigue scores (*β* = 4.60; 95% CI: 4.26, 4.95) and (*β* = 3.49; 95% CI: 3.14, 3.83) respectively. Details are presented in Table 3.

**Table 3:** Association of fatigue symptoms with explanatory variables among males.

*N* = 2,751	Mean distribution	Model 1			Model 2			Model 3		
Variable	Mean fatigue score	*β* coef	95% CI	*p*-value	*β* coef	95% CI	*p*-value	*β* coef	95% CI	*p*-value
Year										
2018	24.6	Ref			Ref			ref		
2023	25.2	0.60	−0.07, 1.28	0.08	0.61	−0.09, 1.30	0.09	0.03	−0.56, 0.62	0.92
Age										
13	24.5	Ref			Ref			Ref		
14	25.0	0.52	−0.43, 1.47	0.29	0.46	−0.51, 1.43	0.35	0.44	−0.36, 1.24	0.28
15	24.8	0.30	−0.63, 1.24	0.53	0.05	−0.91, 1.01	0.92	0.47	−0.33, 1.26	0.25
16–17	25.8	1.34	0.20, 2.48	0.02	0.86	−0.30, 2.02	0.15	0.92	−0.06, 1.90	0.07
City										
Salo	25.7	Ref			Ref			Ref		
Rovaniemi	24.2	−1.53	−2.20, −0.85	<0.001	−1.45	−2.14, −0.75	<0.001	−1.19	−1.78, −0.61	<0.001
Family structure										
Two biological parents	24.3	Ref			Ref			Ref		
One biological/remarried parents/same sex	27.1	2.81	1.97, 3.65	<0.001	2.85	1.97, 3.73	<0.001	1.55	0.82, 2.27	<0.001
Foster/adoptive/other parents	27.4	3.21	0.83, 5.59	0.01	2.38	−0.26, 5.01	0.08	0.28	−1.61, 2.16	0.77
Birth place										
Born in Finland	24.9	Ref			Ref			Ref		
Born abroad	26.4	1.49	−0.04, 3.02	0.06	0.59	−1.00, 2.19	0.47	0.25	−1.13, 1.63	0.73
BMI^†^										
Normal	23.7	Ref			Ref			Ref		
Underweight	25.0	1.32	−0.86, 3.50	0.24	1.24	−1.01, 3.50	0.28	−0.37	−2.23, 1.49	0.70
Overweight	27.2	3.53	2.73, 4.33	<0.001	3.43	2.63, 4.23	<0.001	1.99	1.30, 2.68	<0.001
Psychopathology^‡^										
Internalizing problems		4.60	4.26, 4.95	<0.001	—	—	—	3.74	3.37, 4.11	<0.001
Externalizing problems		3.49	3.14, 3.83	<0.001	—	—	—	2.19	1.85, 2.53	<0.001

Model 1: Adjusted for year.

Model 2: Adjusted for year, age, city, family structure, birthplace.

Model 3: Adjusted for year, age, city, family structure, birthplace, internalizing problems, externalizing problems.

Missing numbers by variable: fatigue 0; year 0; age 25; city 0; BMI 250; family structure 17; birth place 13; internalizing 12; externalizing 12.

† BMI categorization: Boys (Underweight <15.9, Normal 15.9–22.4, Overweight >22.4).

‡ Psychopathology: internalizing and externalizing standardized with a standard deviation of 1.

After adjusting for sociodemographic variables, the increase in fatigue was not significant in 2023 (*β* = (0.61; 95% CI: −0.09, 1.30). Age was not statistically significant while residing in Rovaniemi was associated with lower fatigue (*β* = −1.45; 95% CI: −2.14, −0.75). Compared to living with two biological parents, a family structure comprising of one biological or remarried biological parent was associated with higher fatigue (*β* = 2.85; 95% CI: 1.97, 3.73), while overweight boys had higher fatigue (*β* = 3.43; 95% CI: 2.63, 4.23).

Congruently, the increase in fatigue in 2023 was not significant after adjusting for sociodemographic and mental health variables (*β* = 0.03; 95% CI: −0.56, 0.62) in Model 3. Boys residing in Rovaniemi had lower fatigue (*β* = −1.19; 95% CI: −1.78, −0.61), whereas a family structure comprising of one biological or remarried biological parent was associated with higher fatigue (*β* = 1.55; 95% CI: 0.82, 2.27). Overweight boys had higher fatigue (*β* = 1.99; 95% CI: 1.30, 2.68). Additionally, internalizing and externalizing problems were associated with higher fatigue (*β* = 3.74; 95% CI: 3.37, 4.11) and (*β* = 2.19; 95% CI: 1.85, 2.53) respectively.

## Discussion

This study estimates the changes in fatigue symptoms among Finnish adolescents in the general population before and after the pandemic. Our study is the first, to our knowledge, to estimate the prevalence of fatigue symptoms among adolescents before and after the COVID-19 pandemic.

The first main key finding was the marked increase in the prevalence of fatigue symptoms among female adolescents, rising from 16.2% in 2018 to 24.3% in 2023, while there was no significant change among males (6.7% vs. 5.6%) over the same period. The estimates for girls are comparable to the pooled prevalence of 20.5% reported across four studies, and higher than the prevalence observed among boys in our sample (10). Considering that the pooled prevalence reflects combined sexes, however, our findings are sex-stratified, thus direct comparisons should be interpreted with caution. We found no global nor cross-cultural sex-stratified findings on fatigue among adolescents in the general populations for robust comparisons, indicating a paucity of data on this important aspect of health and well-being. The increase in fatigue symptoms post-pandemic suggests that the societal changes and disruptions caused by COVID-19 may have disproportionately affected females, potentially exacerbating existing stressors such as school pressure, social isolation, and lifestyle changes. Alanko et al. found female adolescents faced greater difficulties attending school after the pandemic (39). The possibility that the fatigue symptom levels reflected the adolescents' immediate life situations, hobbies, or academic pressures, rather than the lingering effects of the pandemic cannot be ruled out. On the other hand, the increase in fatigue could be associated with COVID-19 infection; fatigue being one major manifestation of long COVID (or PASC, postacute sequelae of SARS CoV-2) (40, 41). Among long COVID patients, mood disturbances aggravate sleep problems, which is linked to fatigue (40). Approximately 20% of all COVID-19 infections occurred in children and adolescents and 10%–25% of the children suffered from long COVID, with fatigue manifesting in 10%–82% of cases (19, 42–45). It is possible that the rise in fatigue in 2023 could be attributed to the lingering effects of COVID-19 infections considering data collection was conducted slightly before the public health emergency was lifted in May 2023. In Finland, approximately 15.4% of 10–20 year-olds suffered from COVID-19 in 2022 (33, 46). It is tempting to suggest that fatigue alone may be a symptom of mild long COVID. It is also possible that the seasonal variations contributed to fatigue, due to significant fluctuations in temperature during spring time considering the time of the survey data collection. A previous Finnish study found slightly higher irritability and tiredness among adolescents in the northern and southern regions in spring vs. summer months (31). However, the cross-sectional nature of the study, which captures fatigue over a two-week period, limits the ability to draw direct causal inferences about the long-term effects of COVID-19 on fatigue.

A finding that females were more fatigued compared to males is consistent with previous studies (2–4, 47). The prevalence rates observed in this study are comparable to those reported in the Netherlands where 21% of females and 7% of males experienced fatigue (4). However, the mean fatigue scores in our study were higher than the estimates reported in the Dutch study, particularly for males, suggesting that adolescents in Finland may experience a slightly greater burden of fatigue symptoms (4). This could be attributed to regional differences in lifestyle, cultural norms, or educational demands that impact fatigue symptom reporting. Additionally, the same validated scale (CIS) was used in both studies, ensuring methodological consistency in fatigue measurement (4, 23). Interestingly, female sex is a risk factor for long COVID and chronic fatigue syndrome/myalgic encephalitis (CSF/ME) (41, 44, 48–52). Pandemic data show a marked rise in new CFS/ME diagnoses, for which fatigue is a major symptom (53). Emerging biomarker studies reveal sex-specific immune changes in post-infectious fatigue (54), which implies that the higher fatigue burden observed among girls may reflect a combination of psychosocial stressors and biological vulnerability.

In comparison with studies that used different fatigue scales, longitudinal studies from the UK study revealed a prevalence of 34% and 38% at follow up 2 months apart among adolescents in 1999 (2), and at 11% in 2001% and 17% during follow up in 2003 (55). Sommers et al. revealed a prevalence of severe fatigue at 4.4% in 2019/2020 among German adolescents, which was lower than this study (3). However, the results may not be directly comparable to our study which used 2 separate cross-sectional surveys at the two time points. A study from Japan found sex was not associated with fatigue (13). This discrepancy could be attributed to cultural differences in gender roles, social expectations, and the stigma associated with reporting fatigue or other health issues, especially among males. In some cultures, males may be less likely to report fatigue symptoms, potentially skewing the prevalence. These sociocultural factors underscore the importance of considering the broader context when interpreting findings related to fatigue. Among US adolescents, fatigue was prevalent at 21% in 1994/1995 (56), prolonged fatigue at 3% in 2001/2004 (47) and morning fatigue at 30.6% among females in 1997/1998 (12). Some of the variation may arise from differences in scale (e.g., morning vs. general fatigue), cut-off thresholds, and survey design (cross-sectional vs. longitudinal), which indicate the need for standardized measures to streamline results and better comparisons across different populations and time.

The second key finding was the association between higher levels of fatigue and increased psychopathology, specifically internalizing and externalizing symptoms. Moreover, females had higher mean scores both for internalizing and externalizing problems in this study. A Finnish study found an increase in internalizing problems among female adolescents during the COVID pandemic (57). Fatigue is not only a common symptom of mental health disorders, such as anxiety and depression, but may also serve as a precursor to or consequence of these conditions (3, 4, 58). Approximately half of adolescents experiencing prolonged fatigue symptoms also report co-morbid anxiety or depression in one study (47). This co-occurrence is not unique to adolescents; studies in adult populations report that between 44% and 75% of fatigued individuals also suffer from depression or anxiety (47). The close connection between fatigue and psychopathology raises important clinical implications. This relationship is further complicated by the fact that mental health problems often begin during adolescence, a developmental period characterized by heightened sensitivity to stressors, social pressures, and academic challenges. Females, in particular, are more likely to report experiencing stress, anxiety, and depression, which may explain the higher levels of fatigue symptoms observed (4, 47). Moreover, studies suggest that females are more likely to internalize stress and emotional distress, which can manifest as fatigue, while males may engage in externalizing behaviors, such as aggression or hyperactivity, which are less likely to be associated with fatigue (59). Additionally, females may be more attuned to their physical and emotional states, leading to a higher likelihood of reporting fatigue symptoms. These findings highlight the importance of sex-sensitive approaches to mental health and fatigue management, particularly in adolescent populations.

### Limitations

One of the primary limitations is the cross-sectional design, which limits the ability to infer causal relationships between the COVID-19 pandemic and the reported increase in fatigue symptoms. Additionally, the post COVID survey was conducted in 2023 when the pandemic was less impactful. Another limitation is the reliance on self-reported data, which introduces the potential for recall bias. Self-report surveys are subject to recall bias, where participants may inaccurately remember or report their fatigue levels, especially when asked to reflect on symptoms over a two-week period. This is particularly relevant in adolescent populations, where memory recall and self-awareness may vary widely. Additionally, we did not have information about COVID-19 infection history among the study participants; thus, determining fatigue symptoms directly related to the infection and long COVID was not possible. Furthermore, we did not ask for other common symptoms for long COVID (e.g., headache or respiratory symptoms) or for CSF/ME (post-exertional malaise or unrefreshing sleep). Furthermore, it was possible that the samples in 2018 and 2023 may not be comparable, however, we ensured the samples were similar by inviting adolescents from the same schools, classes and age to ensure comparability across time.

Despite the limitations, the study has significant strengths. One of the primary strengths of this study is its unique pre- and post-COVID design, making it one of the first to assess fatigue levels among adolescents in the general population before and after the COVID-19 pandemic. To our knowledge, no prior research has directly compared fatigue in adolescents across these two time periods, providing valuable insight into how the pandemic may have influenced fatigue within this population. This longitudinal aspect, though using two cross-sectional datasets, allows us to explore trends in fatigue in response to a major global event, contributing important evidence to the field of adolescent health. Additionally, the study's inclusion of two medium-sized cities, Rovaniemi and Salo, located in northern and southern Finland, enhances the generalizability of the findings in Finland. These cities represent both urban and rural communities, and their demographic characteristics closely mirror those of the broader Finnish adolescent population.

## Conclusion

The findings of this study have important practical implications for addressing adolescent fatigue, particularly in the context of post-pandemic recovery. The increased prevalence of fatigue symptoms among females may be linked to internalizing problems, and societal changes which highlights the need for mental health support and stress management. Schools, healthcare providers, and policymakers should prioritize programs that promote emotional and physical well-being and resilience, especially targeting female adolescents who are at greater risk of internalizing problems, which is linked to fatigue. Public health initiatives could consider promoting balanced routines that address academic, social, and extracurricular pressures, fostering an environment that supports physical and emotional well-being.

## Data Availability

The raw supporting the conclusions of this article may be made available by authors through contacting the research group.
